# “Two zones and three centers” distribution and suitable areas shift of an evergreen oak in subtropical China under climate scenarios

**DOI:** 10.1002/ece3.70300

**Published:** 2024-09-11

**Authors:** Jinkai Zhang, Song Huang, Jiaxiang Li, Lingjuan Liao, Xiaolong Jiang, Yongfu Xu, Xunlin Yu, Lei Wu, Lijuan Zhao, Jin Fu, Yun Yang, Chunhua Chen

**Affiliations:** ^1^ College of Forestry, Central South University of Forestry and Technology Changsha Hunan China; ^2^ The Forestry Department of Hunan Province Changsha Hunan China

**Keywords:** Biomod2, climatic niche, geographic distribution, habitat suitability, *Quercus oxyphylla*, species distribution model

## Abstract

Understanding the impact of climate change on the geographical distribution of species is a fundamental requirement for biodiversity conservation and resource management. *Quercus oxyphylla*, an evergreen oak endemic to China, plays a crucial role in maintaining the ecological stability in subtropical regions and high economic value attributed to its dark and high‐density heartwood, but the existing resources are close to endangered. Currently, limited knowledge exists regarding its distribution and potential influences of climate change on suitable areas. This study utilized 63 occurrence records and Biomod2 platform, to predict changes in suitable areas for *Q. oxyphylla* under future climate change. The results revealed that (1) *Q. oxyphylla* showed a pattern of three disjunctive geographical centers in the eastern subregion of subtropical evergreen broad‐leaved forest region (IV_A_): Qinling‐Daba Mountains, Nanling Mountains and Wuyi Mountains center. Currently, the highly suitable areas concentrated in two zones divided by the Yangtze River, that is, the northern subtropical evergreen and deciduous broad‐leaved forest zone (IV_Aii_) and the mid‐subtropical evergreen broad‐leaved forest zone (IV_Ai_). (2) The temperature‐related variables, such as annual temperature range (Bio7), the mean diurnal range (Bio2), and annual mean temperature (Bio1), were identified as the key determinants of the distribution pattern. Because of its considerable climatic variations in temperature and water conditions, *Q. oxyphylla*'s habitat displayed a wider climate niche and strong physiological tolerance to climate change. (3) Under future climate scenarios, the suitable area of the species was expected to overall expand with significant regional differences. The suitable area in IV_Ai_ was expected to expand significantly northward while that in IV_Aii_ was expected to gradually shrink. To address the impact of climate change, it is necessary to develop conservation plans focused around the three distribution centers, implement localized and regional conservation policies, and conduct educational outreach among local people.

## Graphical abstract

This study forecasts the future distribution of *Quercus oxyphylla*, an endangered evergreen oak native to China, under climate change scenarios using Biomod2 and 63 occurrence records. Findings indicate that temperature variables significantly influence its distribution across three disjunctive geographical centers, with suitable areas expected to expand, especially northward in the subtropical evergreen broad‐leaved forest region, underscoring the need for targeted conservation strategies around these centers to mitigate climate change impacts.

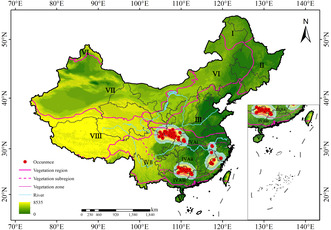

## 1 INTRODUCTION

Climate change has affected ecosystems worldwide, and its consequences are becoming increasingly evident (Huang et al., 2019; Parmesan, 2006). Global warming has intensified since the Industrial Revolution due to the emission of greenhouse gases. The IPCC assessment report showed that the global average surface temperature will increase by a minimum of 0.3–1.7°C to a maximum of 2.6–4.8°C over the 21st century, and the precipitation pattern will also change significantly (Lee et al., 2023; Thackeray et al., 2022). Disruptions in species distributions and ecosystem functions are anticipated as a result of these climatic shifts (Hama & Khwarahm, 2023; Parmesan et al., 2022). Understanding how different species respond to climate change has become a critical focus for the scientific community (Brown et al., 2016; Chmura et al., 2011). Some species are facing severe threats, including decline, endangerment, or even extinction due to climate change (Peng et al., 2021; Subedi et al., 2024), while others may adapt to climate change due to their strong ecological tolerance, thereby expanding their suitable areas (Bellard et al., 2012). These varied responses are often linked to species' the evolutionary histories and ecological niches, which are closely related to climate sensitivity (Yu et al., 2019). Therefore, understanding how plants respond to climate change and the underlying mechanisms is crucial for biodiversity conservation and addressing climate change.

Species distribution models (SDMs) have emerged as pivotal tools in conservation biology, ecology, and biogeography, as they enable the prediction of a species' geographic distribution based on a suite of environmental variables (Blair et al., 2022; Fois et al., 2018). These models serve as a powerful instrument for deciphering the intricate connections between species and their environmental contexts. Typically, SDMs entail the correlation of a species' presence or abundance with an array of environmental parameters, such as climate, topography, soil composition, and vegetation type (Norberg et al., 2019; Parmesan et al., 2022). Through the analysis of these correlations, SDMs can pinpoint the most influential environmental determinants of a species' distribution and forecast its potential range across various scenarios, including those influenced by climate change (Norberg et al., 2019; Subedi et al., 2024). The insights generated by SDMs are invaluable for conservation initiatives, aiding in the identification of areas of paramount conservation significance and the anticipation of the effects of climate change on species ranges. Among the most frequently employed SDMs are maximum entropy (MaxEnt), generalized linear models (GLMs), random forest (RF), artificial neural network (ANN), one rectilinear envelope similar to BIOCLIM (SRE), and flexible discriminant analysis (FDA) (Subedi et al., 2024; Suicmez & Avci, 2023). Despite their constraints, SDMs have revolutionized our understanding of species distributions and have become an essential tool in the quest to conserve and manage biodiversity in the face of environmental change.

*Quercus oxyphylla* Hand.‐Mazz. (Handel‐Mazzetti, 1929), which is an evergreen tree endemic to subtropical China, belongs to Fagaceae and is included in the List of National Key Protected Wild Plants in China (version 2021). The tree is long‐lived and grows in mixed mesophytic forests from 200 to 2900 m above sea level in the subtropical regions of China (Fang et al., 2009; Wu et al., 1999). It was often historically regarded as “miscellaneous wood” and was unknowingly harvested for an extended period, leading to a lack of understanding of this species (Sun et al., 2020; Zhang et al., 1995). In recent years, based on field investigations and wood property studies, we have recognized that this species has favorable ecological functionality, strong adaptability, and high economic value. Its heartwood exhibited a reddish‐brown coloration and dense timber. Concurrently, we have also understood that its natural habitat has suffered significant disturbances and degradation due to anthropogenic activities such as cultivation, grazing, deforestation, afforestation, and tourism (Sun et al., 2021). Encroachment, fragmentation, and habitat eradication have resulted in drastic declines in wild populations (Xie et al., 2021). Therefore, mitigating this threat and implementing measures to restore the conditions necessary for habitat and population recovery are imperative. However, the lack of understanding of its distribution patterns and the key environmental factors that regulate its growth presents obstacles to conservation and management efforts.

In this paper, we aimed to (1) assess the current distribution of *Q. oxyphylla* and identify the dominant climatic variables influencing it, (2) explore how the suitable area of this species might shift under future climate change scenarios. Given the extensive latitudinal and altitudinal range of this species in subtropical China (Wu et al., 1999), we hypothesized that its distribution will expand in response to future climate change.

## 2 MATERIALS AND METHODS

### 2.1 Samples and species occurrence records

**FIGURE 1:**
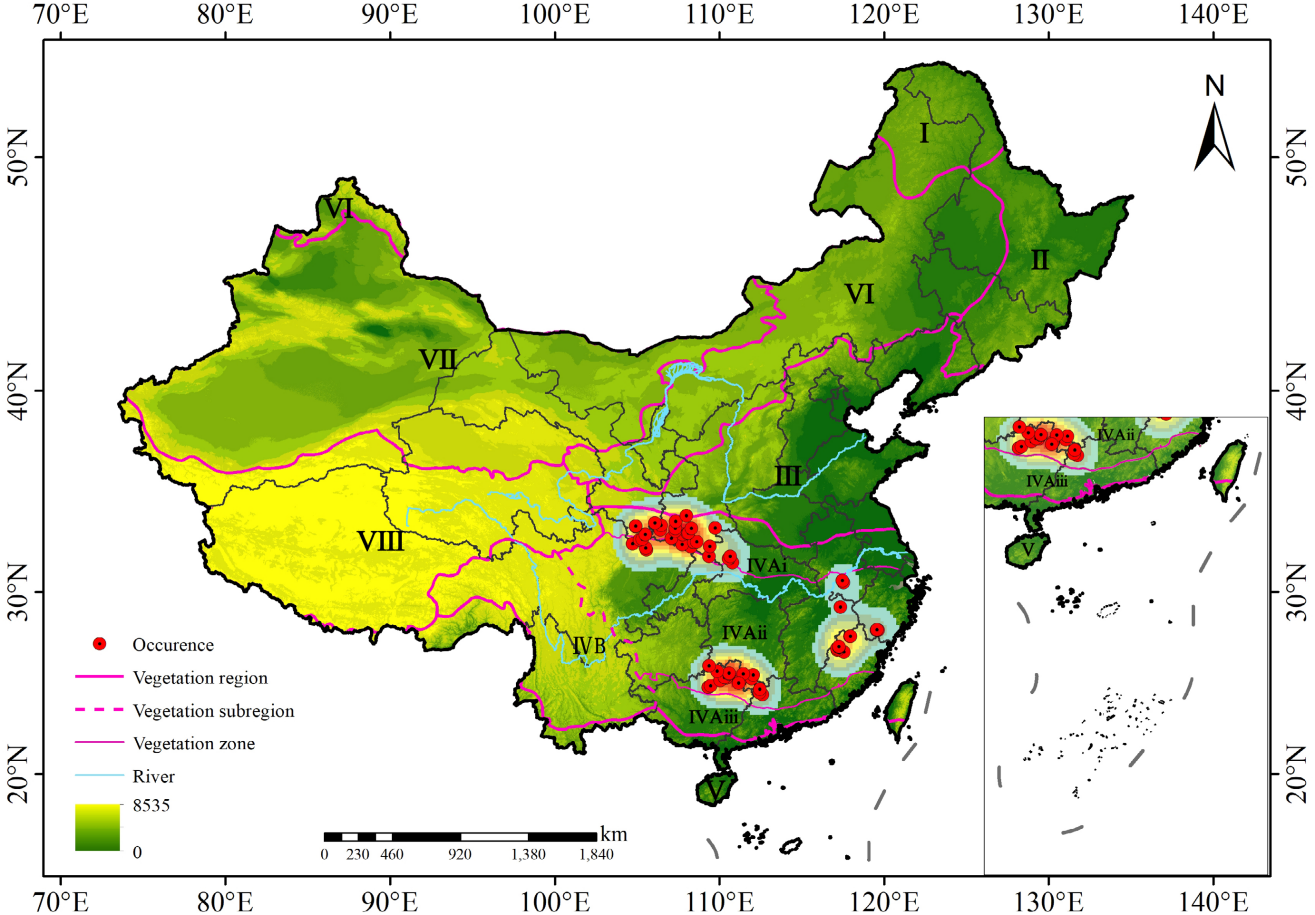
*Q. oxyphylla* occurrence and distribution pattern in China. The roman numerals are used for the vegetation region number. I. Boreal Coniferous Forest Region; II. Temperate Coniferous and Broad‐leaved Mixed Forest Region; III. Warm Temperate Deciduous Broad‐leaved Forest Region; IV. Subtropical Evergreen Broad‐leaved Forest Region, IV_A_. Eastern (Humid) Evergreen Broad‐leaved Forest Subregion, IV_Ai_. Northern Subtropical Evergreen and Deciduous Broad‐leaved Mixed Forest Zone, IV_Aii_. Mid‐subtropical Evergreen Broad‐leaved Forest Zone, IV_Aiii_. Southern Subtropical Monsoon Evergreen Broad‐leaved Forest Zone, IV_B_. Western (Semi‐humid) Evergreen Broad‐leaved Forest Subregion; V. Tropical Monsoon Forest and Rainforest Region; VI. Temperate Grassland Region; VII. Temperate Desert Region; VIII. Qinghai–Tibet Plateau Alpine Vegetation Region.

Data on the geographical distribution of *Q. oxyphylla* were mainly acquired from current population and historical collections, representing 220 records from 86 locations. Data were retrieved from the *Chinese Virtual Herbarium* (CVH, http://www.cvh.ac.cn/), *National Specimen Information Infrastructure* (NSII, http://www.nsii.org.cn/), *Subject Database of China Plant* (http://www.plant.csdb.cn/), *Plant Photo Bank of China* (http://ppbc.iplant.cn/) and relevant published literature (Guo et al., 2019). A taxonomic filtering was applied to all collected data to ensure accuracy and eliminate redundancy. Upon re‐examination, we found that certain specimens previously believed to be from Yunnan, western Sichuan, central Guangxi, and eastern Fujian were misidentified (https://doi.org/10.5061/dryad.qz612jmqn), which indicated that previous publications using these records to delineate the geographical distribution of *Q. oxyphylla* might be inaccurate. After eliminating the misidentified data and incorporating newly occurrence records from our team's fieldwork in recent years, a total of 63 valid occurrence records were obtained encompassing 55 specimen records from 17 herbariums, 4 photography records, 3 field records, and 1 reference record (Figure 1).

The base map used for the analysis was derived from the National Geospatial Information System of China (http://nfgis.nsdi.gov.cn). Using ArcGIS 10.8, kernel density analysis was employed to identify the contemporary distribution center (Figure 1). By overlaying the 1:1,000,000 vegetation zoning map of China (Zhang, 2007), which effectively represents species–climate relationships, we were able to ascertain the general correlation between the geographical distribution patterns and climate. All the maps were georeferenced using the WGS84 coordinate system.

### 2.2 Bioclimatic variables

A total of 19 bioclimatic variables (Table 1) were downloaded from the WorldClim database (version 1.4) (http://www.worldclim.org/) with a spatial resolution of 30 arcseconds, or approximately 1 km^2^ per pixel (Fick & Hijmans, 2017). These variables were used to define the environmental niche of a species, utilizing both present and projected climate data (Hijmans et al., 2005). The time frame for the current period spanned from the 1960–1990s, while the designations of the 2050s and the 2070s refer to the 2041–2060s and 2061–2080s, respectively. The forecasted climate data were aligned with the most recent updates from the 5th IPCC Report. For *Q. oxyphylla* in China, the Climate System Model (BCC–CSM 1.1) was utilized to estimate future climate impacts based on *Representative Concentration Pathway* (RCPs) scenarios RCP2.6, and RCP8.5, which represent low‐, and high‐emission scenarios. These were evaluated during the 2050s and the 2070s (Xie et al., 2021).

**TABLE 1:** Descriptive statistics of 19 bioclimatic variables for 63 occurrence sites of *Q. oxyphylla*.

Code	Variables (unit)	Mean	SD	Min	Max	CV
**Bio1**	**Annual mean temperature (°C)**	**16.01**	**3.02**	**7.80**	**20.80**	**18.89**
**Bio2**	**Mean diurnal range (°C)**	**8.64**	**0.61**	**7.50**	**10.40**	**7.01**
**Bio3**	**Isothermality (%)**	**28.21**	**1.97**	**23.00**	**35.00**	**6.98**
Bio4	Temperature seasonality	755.65	52.27	657.40	873.00	6.92
Bio5	Max temperature of warmest month (°C)	30.86	2.62	23.40	33.90	8.49
Bio6	Min temperature of coldest month (°C)	0.70	3.48	−7.80	6.40	493.91
**Bio7**	**Temperature annual range (°C)**	**30.15**	**1.65**	**27.00**	**34.00**	**5.48**
Bio8	Mean temperature of wettest quarter (°C)	22.63	2.13	16.90	26.70	9.43
Bio9	Mean temperature of driest quarter (°C)	6.97	4.33	−2.80	14.60	62.20
Bio10	Mean temperature of warmest quarter (°C)	25.34	2.58	18.10	28.60	10.19
Bio11	Mean temperature of coldest quarter (°C)	5.86	3.35	−2.80	11.70	57.17
**Bio12**	**Annual precipitation (mm)**	**1269.35**	**426.48**	**507.00**	**1901.00**	**33.60**
Bio13	Precipitation of wettest month (mm)	230.29	73.39	98.00	355.00	31.87
Bio14	Precipitation of driest month (mm)	27.11	20.01	2.00	52.00	73.82
**Bio15**	**Precipitation seasonality (%)**	**66.86**	**11.83**	**48.00**	**98.00**	**17.69**
Bio16	Precipitation of wettest quarter (mm)	611.79	184.00	268.00	933.00	30.08
Bio17	Precipitation of driest quarter (mm)	97.84	70.66	9.00	185.00	72.22
Bio18	Precipitation of warmest quarter (mm)	497.83	112.92	254.00	744.00	22.68
Bio19	Precipitation of coldest quarter (mm)	115.46	88.88	9.00	253.00	76.98

*Note*: Bold indicates variables used for modeling. Abbreviations: CV, coefficient of variability; SD, standard deviation.

In the modeling process, redundant information introduced by strongly correlated variables, which could reduce the reliability and predictability of outcomes, must be excluded (Hundessa et al., 2018). To avoid overfitting between environmental factors (Gebrewahid et al., 2020), Pearson correlation analysis was conducted on 19 climate factors using the “Hmisc” package in R 4.3.2 software (Figure S1). One variable per pair with high correlation coefficient (≥0.8) was removed, resulting in seven climate factors: the annual mean temperature (Bio1), the mean diurnal range (Bio2), the isothermality (Bio3), the temperature annual range (Bio7), the mean temperature of wettest quarter (Bio8), the annual precipitation (Bio12), and the precipitation seasonality (Bio15) (Table 1).

### 2.3 Modeling species distribution

Data on the occurrence of *Q. oxyphylla* and seven selected bioclimatic variables during three periods (current, 2050s, and 2070s) were analyzed using a combined modeling approach and utilizes the six common modeling algorithm options (MaxEnt, GLMs, RF, ANN, SRE, and FDA) supported by the *Biomod2* platform in R (Thuiller et al., 2024). To improve species distribution model accuracy, during model simulation, 1000 pseudo‐presence point was randomly generated based on the distribution range of each species. A random 75% of sample data was used to train the model, with the remaining 25% used for validation. The weights for presence data and pseudo‐presence data were set to be equal. For the six single model algorithms, the models were run with 10 repetitions each, resulting in 60 models. All models with a TSS > 0.70 were screened (Marmion et al., 2009). The model results that met the requirements were then normalized and multiplied by their corresponding weights to be added together. When integrating the models, the single model weights were automatically allocated based on their TSS values (Subedi et al., 2024). We divided the combined model simulation results into: unsuitable areas (0–0.25), low suitable areas (0.25–0.50), medium suitable areas (0.50–0.75), and high suitable areas (0.75–1.00).

### 2.4 Model evaluation

The accuracy of the model prediction was evaluated using the area under the curve of receiver operating characteristic curves (ROC), the true skill statistic (TSS), and Kappa coefficient (Liu et al., 2024). Higher ROC and TSS values indicated a stronger relationship between the species distribution model and environmental variables, suggesting higher model prediction accuracy (Guo et al., 2015). ROC values range from 0.5 to 1, with values between 0.80 and 0.90 indicating high model simulation accuracy, and values between 0.90 and 1.00 indicating extremely high accuracy (Chen et al., 2022). The TSS evaluation metric ranged from −1 to 1, with values between 0.70 and 0.85 indicating good simulation results, and values between 0.85 and 1.00 suggesting extremely good results (Marmion et al., 2009). The Kappa statistic was a measure of how the accuracy of the model's predictions compared with those expected by chance. A Kappa value greater than 0.85 indicated that the model's performance is exceptionally good (Araujo et al., 2005).

### 2.5 Environmental factor importance analysis

Factor importance was used to evaluate the contribution of environmental variables in limiting species' current geographic distribution patterns (Harms et al., 2020). These data relied on a specific pathway for finding the optimal solution, by progressively adjusting the coefficients of individual elements to increase the gain value, then allocating the incremental gain value to the environmental variable that determined the element, and converting it into a percentage before presenting it (Brown, 2014). To analyze the dominant environmental driving variables influencing the suitable distribution of the species under baseline climate, the weight values of each environmental factor from the combined model output by the *Biomod2* package were used (Thuiller et al., 2024), and related boxplots were drawn using the *ggplot2* package.

### 2.6 Suitable range change and centroid migration

A threshold of 0.75 was set to generate a 0–1 raster map for presence/absence. The number of raster cells representing the presence of each species in different time periods was counted. A bar chart representing the change in species area over different periods was generated. Using the SDMtoolboxs toolkit (Brown, 2014) in ArcGIS v10.8, the centroids of suitable areas were calculated based on all presence/absence distribution maps. Vector files were generated showing the direction and magnitude of change in suitable area centroids between adjacent time periods to represent the direction and distance of species centroid migration under different climate scenarios (Hama & Khwarahm, 2023).

## 3 RESULTS

### 3.1 Model performance

**FIGURE 2:**
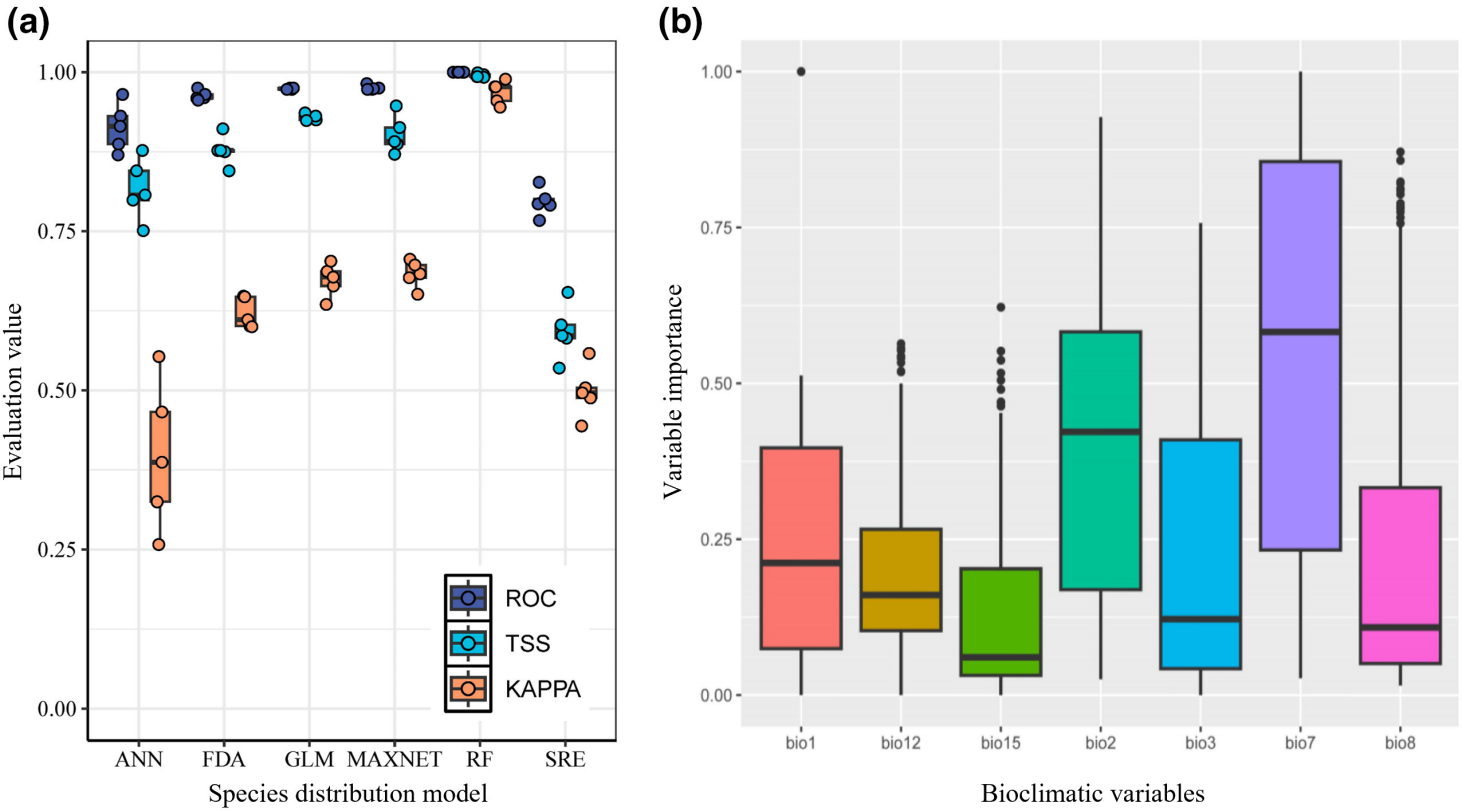
Evaluation results of different models (a) and importance of bioclimatic variables (b).

The simulation accuracy of six models differed greatly. By selecting and combining models that exhibited TSS value above 0.7 in simulation results, the stability and accuracy of the overall model's simulation performance were significantly enhanced (Figure 2a). Among these models, ANN, RF, GLM, MaxEnt, and FDA models demonstrate high simulation accuracy and showed greater consistency in their repeated model analyses. The average TSS and ROC for these models all surpassed the 0.8 threshold. However, most of the results from the SRE did not meet the standards for model integration.

### 3.2 Importance of bioclimatic variables

The results of the combined model's environmental factor importance showed that bioclimate factors related to temperature were more important than those related to precipitation. Specifically, the annual temperature range (Bio7) was the most crucial climate variable influencing the geographic distribution of *Q. oxyphylla*, with its impact far exceeding that of other factors (Figure 2b). The next most important factor was the mean diurnal range (Bio2). Among precipitation‐related factors, annual precipitation (Bio12) was the most significant, while precipitation seasonality (Bio15) was the least significant (Figure 2b).

### 3.3 Distribution pattern under the current climate scenario

The sample data of *Q. oxyphylla* was located between latitudes 24.5° N and 33.9° N and longitudes 104.7° E and 119.6° E, which showed three disjunctive geographical centers in the eastern subregion of subtropical evergreen broad‐leaved forest region (IV_A_): (1) Qinling‐Daba Mountains center at the junction of Shaanxi, Gansu, and Sichuan provinces; (2) Wuyi Mountains center at the junction of Fujian and Zhejiang provinces; (3) Nanling Mountains center at the junction of Guangxi, Guangdong, and Hunan provinces (Figure 1).

**FIGURE 3:**
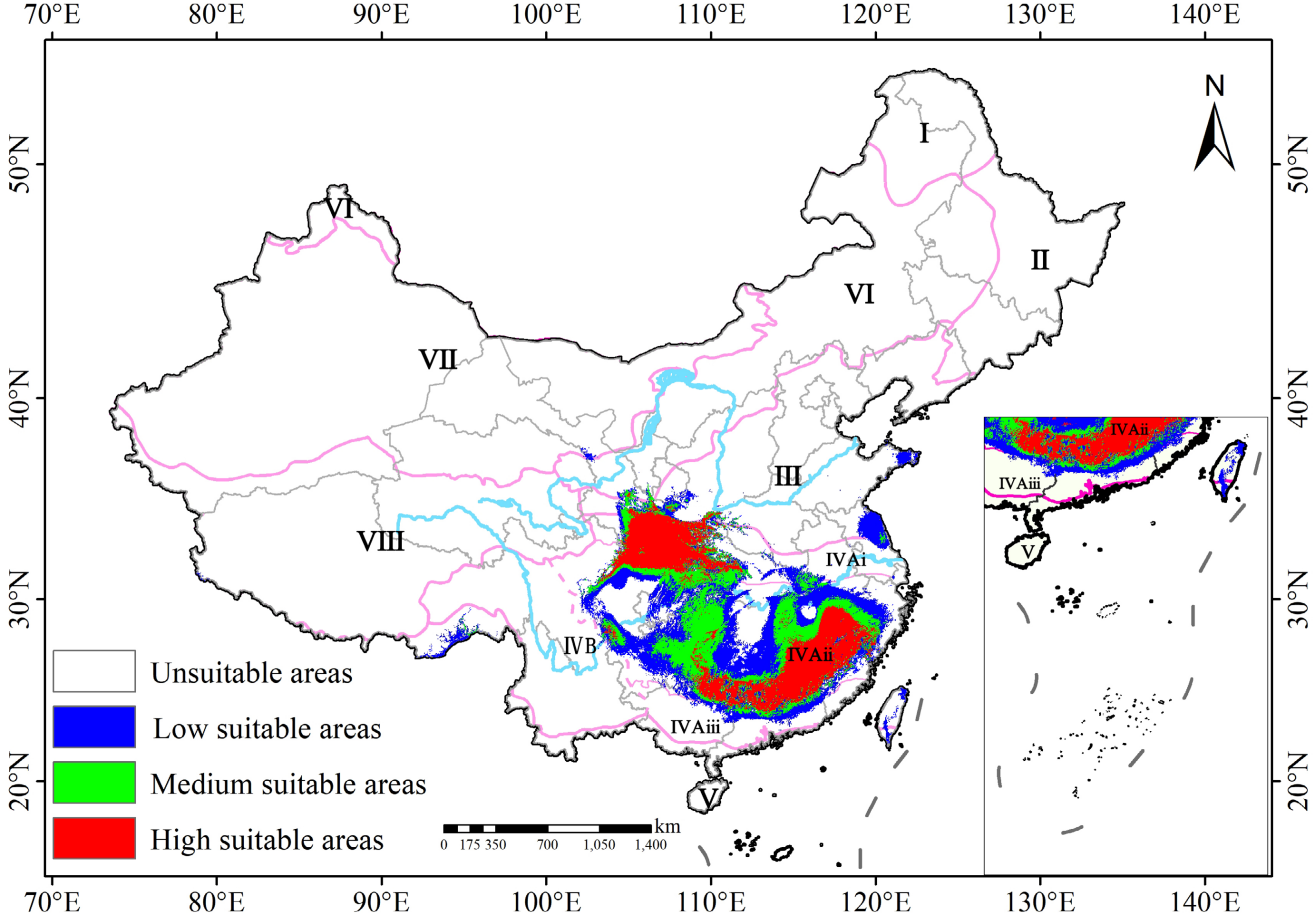
Potential suitable areas of *Q. oxyphylla* under the current climate condition. Red, green, and blue indicate high, medium, and low suitable areas, respectively. The roman numerals are the same to Figure 1.

Under the current climate scenarios, the potentially suitable areas for *Q. oxyphylla* laid approximately between latitudes 24.0° N and 35.0° N and longitudes 102.2° E and 120.8° E, far larger than the current actual range (Figure 3). The highly suitable areas exhibited a distinct discontinuous distribution pattern, concentrated in two zones: (1) North of the Yangtze River, covering the Qinling‐Daba Mountains in the northern subtropical evergreen and deciduous broad‐leaved forest zone (IV_Aii_). (2) South of the Yangtze River, including the Nanling Mountains and Wuyi Mountains in the mid‐subtropical evergreen broad‐leaved forest zone (IV_Ai_). Between these two regions, the Wuling Mountains, Xuefeng Mountains, and Wushan Mountains were primarily moderately suitable areas. The hilly areas surrounding the Sichuan Basin, Dongting Lake, and Poyang Lake were predicted low suitability regions (Figure 3).

### 3.4 Future distribution under climate changes

**FIGURE 4:**
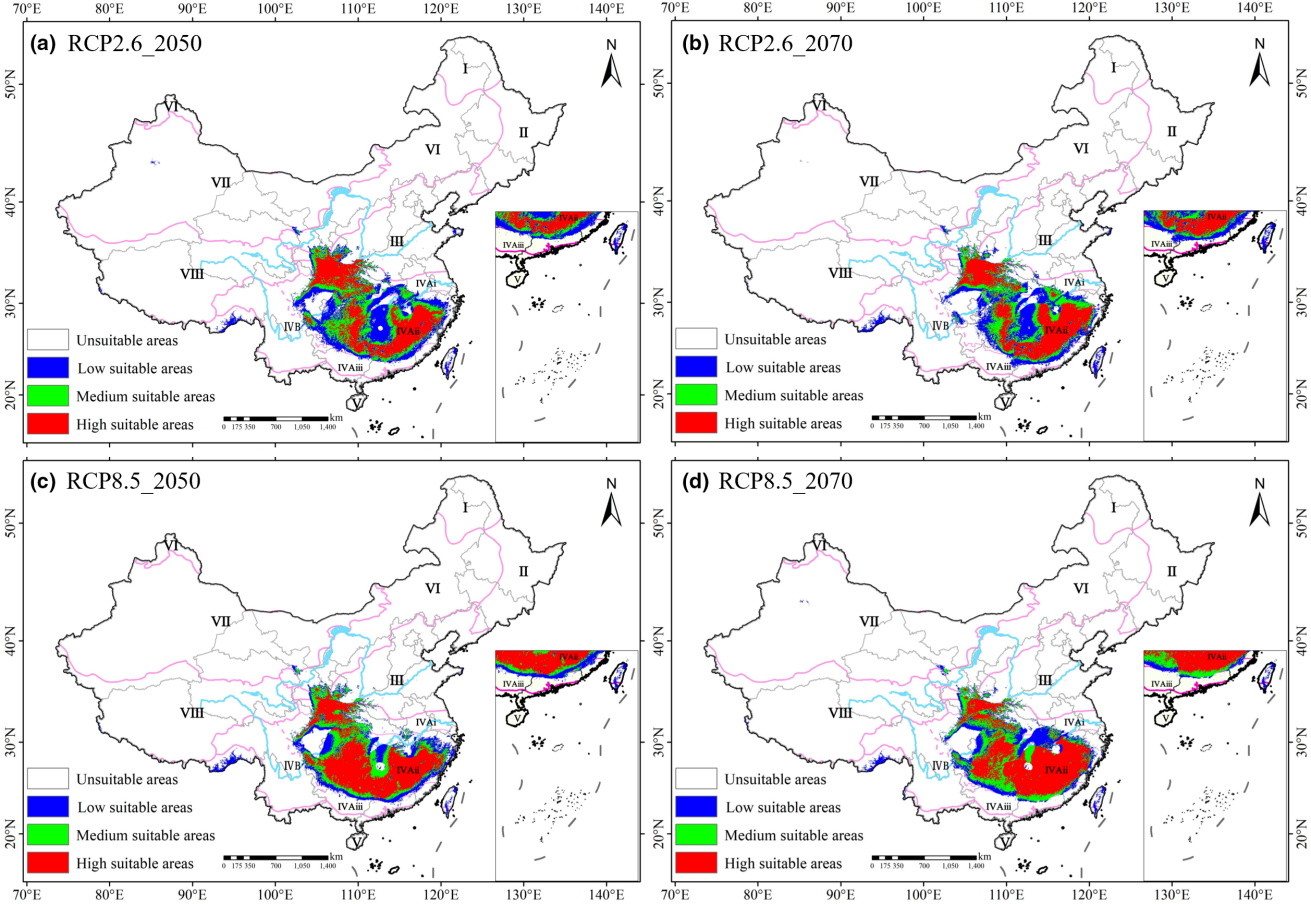
Projected suitable area for *Q. oxyphylla* by 2050s and 2070s under the RCP 2.6 (a, b) and RCP 8.5 (c, d) climate scenarios. The roman numerals are the same to Figure 1.

**FIGURE 5:**
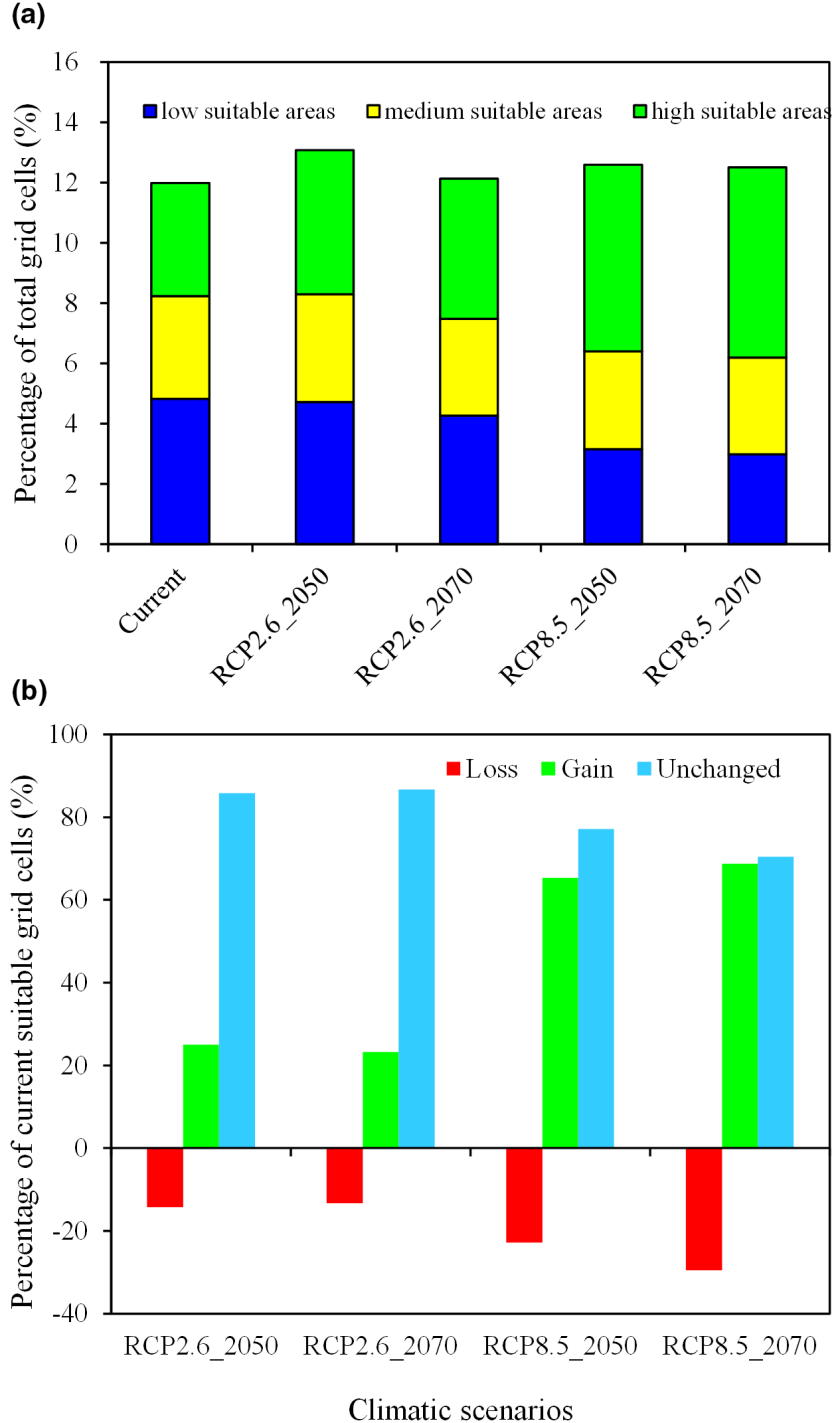
Projected suitable area changes for *Q. oxyphylla* by 2050s and 2070s under the RCP 2.6 and RCP 8.5 climate scenarios. (a) indicates the percentage of different suitable grid cells out of the total predicted grid cells. (b) indicates the percentage of grid cell changes relative to current climate suitable area.

Under future climate change scenarios, the total suitable areas of *Q. oxyphylla* displayed similar increasing magnitudes and trends, with a notable increase in the 2050s, followed by slightly decrease in the 2070s (Figure 4). Compared with the current suitable areas (11.98% of total grid cells), they shared 13.08% (RCP2.6_2050) and 12.13% (RCP2.6_2070) of total grid cells, 12.59% (RCP8.5_2050), and 12.50% (RCP8.5_2070) (Figure 5a).

Overall, the predicted suitable areas for *Q. oxyphylla* were concentrated in the IV_Ai_ and IV_Aii_ vegetation zones of the eastern subregion of the subtropical evergreen broad‐leaved forest. With the Yangtze River as a boundary, the suitable areas in the Qinling‐Daba mountains north of the river gradually decreased, while the south of the river significantly increased (Figure 4). As climate warming intensifies over time, the number of grid cells in low and moderate suitability areas would gradually decrease, while the range of high suitability areas significantly would increase (Figure 5a).

### 3.5 Suitable areas overlap and centroid shift

**FIGURE 6:**
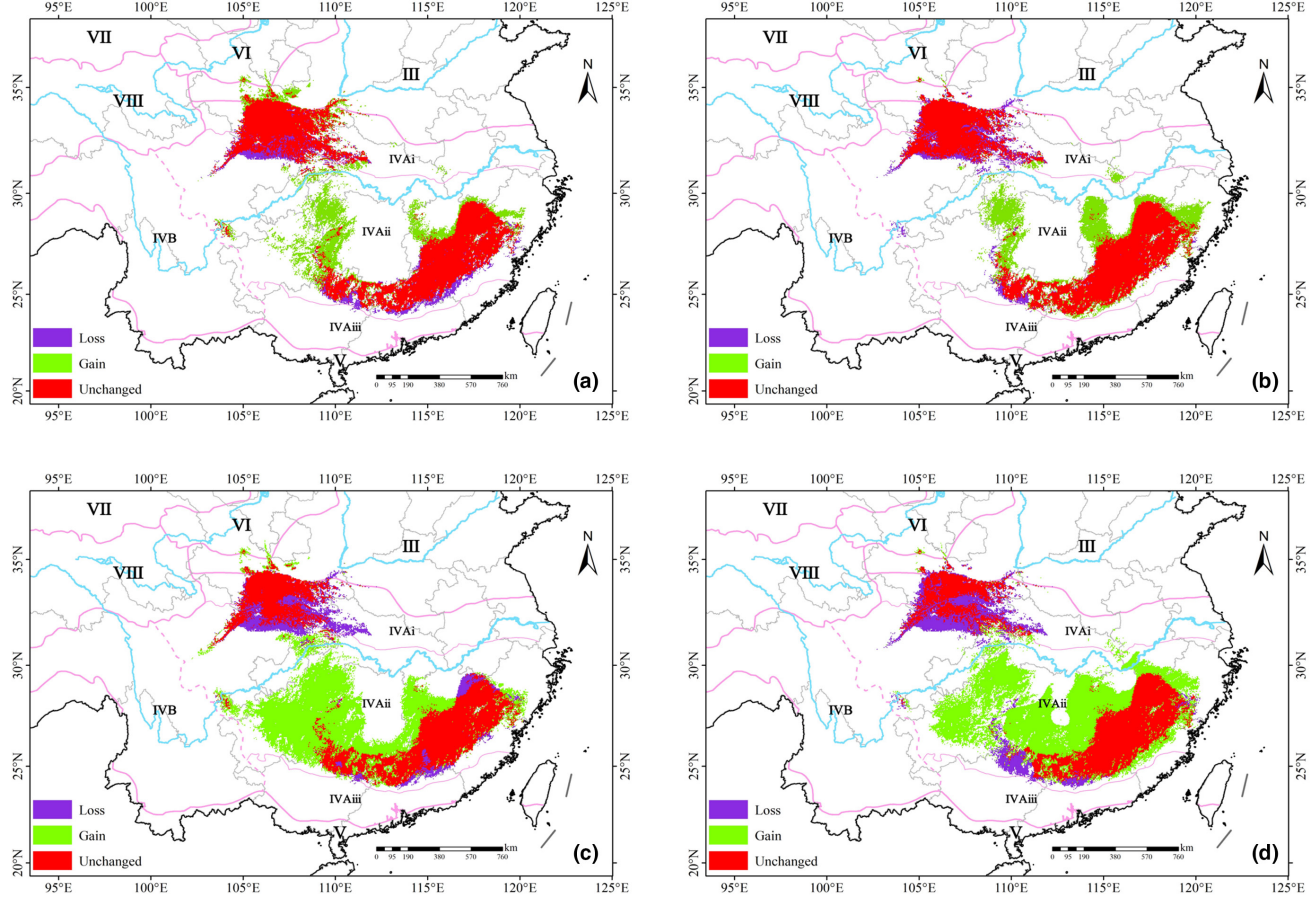
Projected suitable area change for *Q. oxyphylla* by 2050s and 2070s under the RCP 2.6 (a, b) and RCP 8.5 (c, d) climate scenarios.

Under projected future climate scenarios, the suitable range of *Q. oxyphylla* would generally expand outward in response to global warming (Figure 6). However, distinct trends emerged under different climate scenarios and across geographical regions. Under the less severe RCP2.6 scenario, the expansion trend showed a slight weakening over time. Conversely, under the more extreme RCP8.5 scenario, the expansion trend intensified slightly (Figures 5b and 6).

Geographically, a clear north–south divide was observed (Figure 6). South of the Yangtze River, *Q. oxyphylla* exhibited a significant northward expansion, accompanied by a northeastward shift, spreading from the Yuecheng Mountains to the Wuling Mountains, and from the Xuefeng Mountains to the Wushan Mountains. Northward expansion was also observed in the Wuyi Mountains, reaching the Mulianjiu Mountains and northeastward. In contrast, north of the Yangtze River, the Qinling‐Daba Mountains witnessed a less pronounced expansion, with the suitable area shrinking over time and with intensifying global warming. This contraction in the Qinling‐Daba Mountains contributed to the overall loss of suitable habitat for *Q. oxyphylla*, which was particularly notable in the southern and eastern parts of the mountain range, as well as along the southern edge of the Nanling Mountains‐Wuyi Mountains.

**FIGURE 7:**
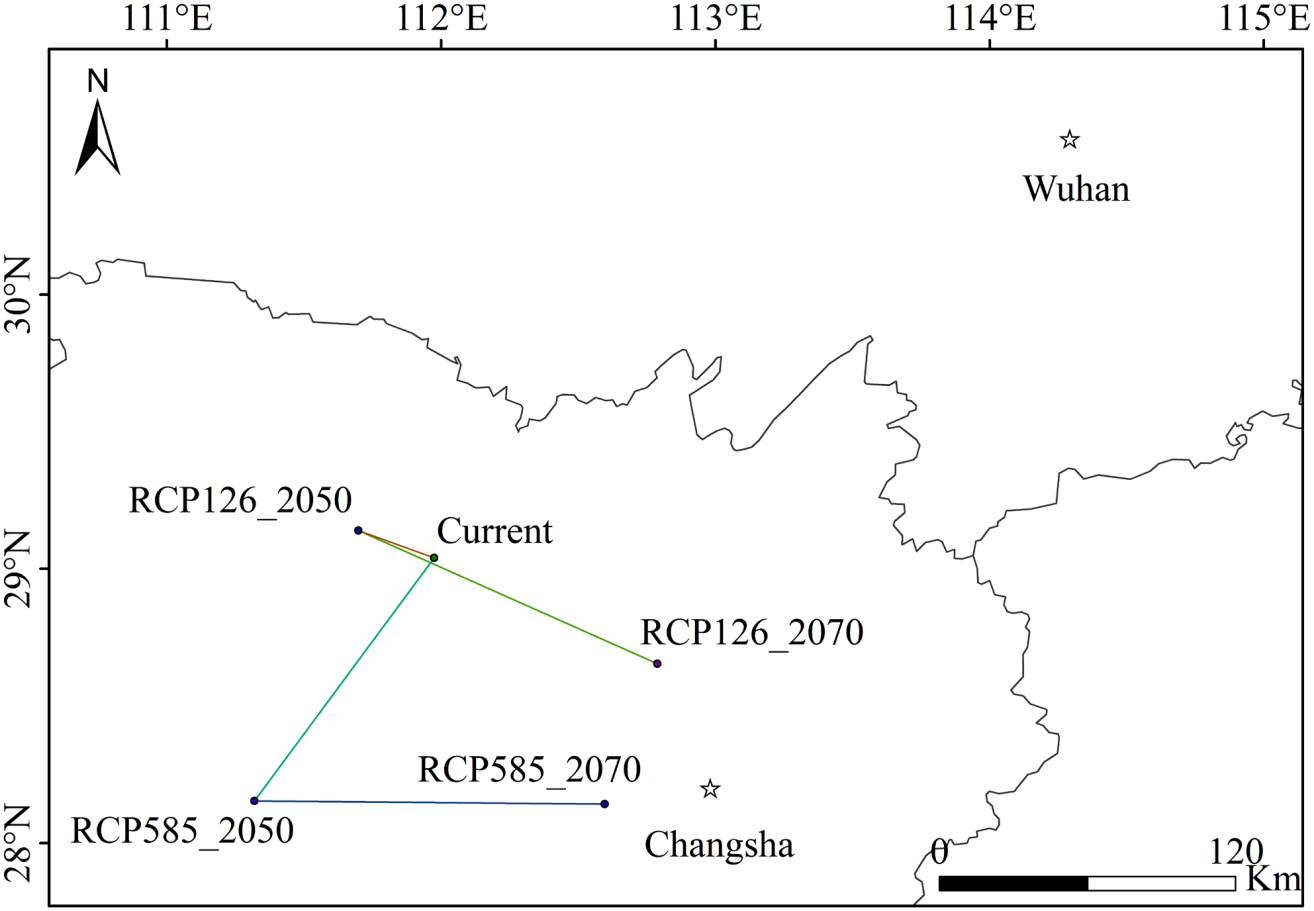
Shifts of centroids of *Q. oxyphylla* under different climate scenarios.

In terms of centroid migration (Figure 7), the centroid of *Q. oxyphylla* was located in Changde, Hunan province. Under the RCP2.6 scenario, by the 2050s, the centroid was projected to shift 35 km west, and by the 2070s, it was expected to move 135 km southeast to Yiyang, Hunan province. In the extreme climate scenario (RCP8.5), the centroid was anticipated to first migrate 135 km southwest to Jishou by the 2050s, and then relocate 150 km east to Changsha by the 2070s. Overall, under future climate scenarios, *Q. oxyphylla* primarily exhibited an eastward migration trend.

## 4 DISCUSSION

### 4.1 Geographical distribution pattern of *Q. oxyphylla*

The occurrence records and current highly suitable areas of *Q. oxyphylla* indicated three disjunctive geographical centers of Qinling‐Daba Mountains, Nanling Mountains, and Wuyi Mountains in the eastern subregion of subtropical evergreen broad‐leaved forest region (IV_A_) (Figures 1 and 3). Interestingly, the mountains where the distribution and suitable areas were located were only in a roughly east–west direction, which coincided with the important geographic dividing region of climate and vegetation zonation in China (Ding, 2013; Zhang, 2007). To the north, the Qinling‐Daba Mountains is located on the northern region of the Chinese subtropics (Yang et al., 2018), which documents the northern subtropical evergreen and deciduous broad‐leaved forest zone (IV_Aii_) (Figure 1). In the south, the Nanling and Wuyi Mountains stand in the southern part middle subtropical evergreen broad‐leaved forest zone (IV_Ai_) (Huang et al., 2023). Synthetically, we suggested that the *Q. oxyphylla* exhibited a disjunctive geographical distribution pattern of “two zones and three centers.”

The spatial patterns of biological species result from contemporary climate, disturbance, and geological history (Simeone et al., 2016). *Q. oxyphylla* belongs to the *Quercus* Group *Ilex*, also known as Eurasian sclerophyllous oaks, whose distribution displays a more or less continuous range in Eurasia, with higher ecological and taxonomic diversity in the Himalayas and adjacent areas (Denk & Grimm, 2010; Zhou, 1992). On the one hand, Zhou's (1992) research on the origin, evolution, and diffusion of *Quercus* in China showed that the group of *Q. oxyphylla* may have originated in the northern section of the Hengduan Mountains and then spread eastward. The Qinling‐Daba, Nanling, and Wuyi Mountains were important channels for the west–east migration of plants in Central China (Wang, 1992). *Q. oxyphylla* migrated eastward along these two channels after its origin and formed a current pattern. On the other hand, the east–west Qinling‐Daba Mountains and the Nanling Mountains were pushed by the Indian Ocean plate in the Eocene, uplifted by remote effects, and stopped forming in the Oligocene (Xu et al., 2016). The north–south trending Wuling Mountains and Xuefeng Mountains experienced intense uplift movements during the Middle and Late Cenozoic. Recent molecular phylogenetic studies have suggested that the split between the evergreen Groups *Cyclobalanopsis* and *Ilex* occurred during the Eocene/Oligocene and that the distribution was constantly affected by frequent crustal movements in the occurrence area (Denk & Grimm, 2009; Simeone et al., 2016). Therefore, we believed that the disjunctive distribution of *Q. oxyphylla* was possibly linked to the high tectonic activity of past and modern distribution ranges, which led to the absence of *Q. oxyphylla* in the mountainous (e.g., the Wuling Mountains and Wushan Mountains) and hilly areas among the three centers. Thus, the “two zones and three centers” pattern reflected geographic area disruptions, which might be closely related to its origins, migration, and geological history.

### 4.2 Current suitable areas and key bioclimatic variables

Under current climatic conditions, the distribution areas of *Q. oxyphylla* is mainly at 200–2900 m altitude between 24° N and 35° N in China (Figure 1). These areas have moderate temperatures and sufficient rainfall in the summer and belong to eastern region of the subtropical evergreen broad‐leaved forest region (Figure 1). Based on the extraction results of climatic factors for each distribution point, in its actual occupied distribution space, Bio1 ranging from 7.8 to 20.8°C, Bio12 from 507 to 1901 mm, Bio7 from 27 to 34°C, and Bio2 from 7.5 to 10.4°C, indicating that *Q. oxyphylla* had a wide climate niche. The large annual temperature difference and wide altitudinal distribution suggested that the habitat of *Q. oxyphylla* had large climate variability and has strong physiological tolerance to climate change, supporting the “climatic variability hypothesis” (Chan et al., 2016; Wang et al., 2023), which also implied that *Q. oxyphylla* might adapt to future climate change due to its wide climate niche.

The results of variable importance analysis verified that the top three bioclimatic variables were temperature factors: Bio7, Bio2, and Bio1, whereas the precipitation factors were relatively less important (Figure 2b). The results were similar to those of other species of *Quercus* in that temperature‐related variables played a key role in controlling their potential biogeographical ranges, such as *Q. fabri*, *Q. mongolica*, and *Q. phillyreodies* (Sun et al., 2020). On the one hand, *Q. oxyphylla* mainly growed in steep mountainous or hilly areas with high rock exposure, indicating it was a drought‐tolerant plant that did not have a high water requirement. In contrast, the leaves of this species were hard and leathery, the lower epidermis and twigs were densely covered with trichomes and had a stronger ability to resist drought than typical mesophytes. Therefore, precipitation was not a key factor restricting the distribution of *Q. oxyphylla*.

### 4.3 Potential adaptability of *Q. oxyphylla* to future climate change

Different species have different mechanisms to adapt to climate change (Dudley et al., 2019). Over time, and with social development, the potentially suitable habitats of species may decrease, remain unchanged, or increase (Feng et al., 2023; Puchałka et al., 2021). Most native plants, including endangered and threatened species, show a trend of shrinking suitable areas, which is closely related to their biological characteristics and inability to shift to suitable habitats (Urban, 2015; Yesuf et al., 2021). Surprisingly, predictions regarding the potentially suitable habitats for *Quercus* mostly show an increasing trend due to their good tolerance, adapting to a variety of environmental conditions (Gimeno et al., 2009; Sun et al., 2020; Wang et al., 2023). Consistently, in this study, the potentially suitable areas of *Q. oxyphylla* were predicted to expand with intensifying climate warming (Figures 3, 4 and 6). This might be attributed to their strong adaptability to temperature and drought conditions. An increase in average temperatures would lead to extreme meteorological events, and the frequency and intensity of extreme weather events (e.g., high temperatures and droughts) in most land areas might continue to increase in the future (He, 2022). As a plant with a broad temperature niche and drought tolerance, *Q. oxyphylla* should be able to adapt to future climatic conditions in eastern area of Chinese subtropics.

Simultaneously, results revealed exhibits distinct adaptability in different geographical regions and climate conditions. Under the backdrop of a warming climate, the high suitable area for the species would increase (Figures 4 and 5a), indicating warming might be conducive to the reproduction and survival of the species. Additionally, there were significant regional differences in the suitable habitat of *Q. oxyphylla* (Figures 4 and 6). In the Qinling‐Daba Mountain region north of the Yangtze River, the suitable habitat area gradually shrinks under climate change, which indicated that climate change could lead to be more vulnerable to threats of the species in the region. Conversely, the region from the Nanling‐Wuyi Mountains region south of the Yangtze River would experience a significant northward expansion. This suggested that the mid‐subtropical evergreen broad‐leaved forest zone (IV_Aii_) was a stable habitat for *Q. oxyphylla*, and future conservation efforts could focus on this area. These findings highlighted the complex and region‐specific impacts of climate change on species distribution, emphasizing the vulnerability of certain regions (Qinling‐Daba mountains) to habitat loss under future climate scenarios (Figure 6).

Furthermore, based on our research findings, developing species‐specific conservation action plans is critical for sustainable conservation of *Q. oxyphylla*. Moreover, to mitigate the risk of local extinctions driven by climate change, a pressing task is to identify suitable areas and establish new populations with high genetic diversity through translocation conservation efforts. Due to the differing responses of northern and southern populations to climate change, habitat conservation for *Q. oxyphylla* demands a focused approach incorporating sustainable local and regional policies, adaptive management strategies, and habitat restoration. Based on our investigations of modern distribution and field surveys, most *Q. oxyphylla* populations are found near villages outside protected areas, primarily in “*Fengshui*” forests or secondary forests. Thus, a priority lies in educating local communities about the importance of the Sharp‐leaved Oak, its habitat, and the impacts of climate change. Furthermore, given the valuable timber resources and broad climatic adaptability of the species, afforestation efforts in climatically suitable regions can contribute positively to the sustainable use of forests under changing climate.

## 5 CONCLUSIONS

As one of the few evergreen oaks in subtropical regions, *Q. oxyphylla* exhibited a unique disjunctive distribution pattern of “two zones and three centers” and a wide altitudinal distribution, indicating its broad climatic niche and ecological tolerance. The climate‐related variables (Bio7, Bio2, and Bio1) were the key climatic factors that determined the distribution pattern, which might also be related to the evolution and migration routes of *Quercus* since the Tethys Sea. As global climate warming intensifies, the distribution of *Q. oxyphylla* would exhibit regional sensitivity and vulnerability. The suitable area in north of the Yangtze River will gradually shrinking. Meanwhile, the suitable area in the south of the Yangtze River would expand northward on a large scale and show an eastward migration trend. These findings provided a scientific basis for developing effective conservation strategies and sustainable resource utilization of the species under climate change. Nonetheless, this study only focused on the species' climatic niche rather than its actual niche. Other factors like species interactions, geographic barriers, and disturbances, might also influence the species' distribution within suitable areas. Consequently, further researches would be needed into the ecological mechanisms of *Q. oxyphylla* population dynamics and habitat changes to provide a scientific foundation for adaptive management.

## AUTHOR CONTRIBUTIONS

**Jinkai Zhang:** Conceptualization (equal); data curation (equal); formal analysis (equal); investigation (equal); methodology (equal); software (equal); visualization (equal); writing – original draft (equal); writing – review and editing (equal). **Song Huang:** Data curation (equal); formal analysis (equal); methodology (equal); software (equal); visualization (equal); writing – review and editing (lead). **Jiaxiang Li:** Conceptualization (lead); formal analysis (equal); methodology (equal); validation (equal); writing – original draft (equal); writing – review and editing (equal). **Lingjuan Liao:** Project administration (equal); supervision (equal). **Xiaolong Jiang:** Methodology (equal); software (equal); writing – review and editing (equal). **Yongfu Xu:** Data curation (supporting); writing – review and editing (supporting). **Xunlin Yu:** Data curation (supporting); writing – review and editing (supporting). **Lei Wu:** Data curation (supporting); writing – review and editing (supporting). **Lijuan Zhao:** Writing – review and editing (supporting). **Jin Fu:** Investigation (equal); writing – review and editing (supporting). **Yun Yang:** Investigation (equal); writing – review and editing (supporting). **Chunhua Chen:** Project administration (equal); supervision (equal).

## FUNDING INFORMATION

This work was funded by the Hunan Provincial Natural Science Foundation (grant number 2023JJ30995), Investigation of Technological Basic Resources of Ministry of Science and Technology of China (grant number 2019FY202300), National Natural Science Foundation (grant number 31901136).

## CONFLICT OF INTEREST STATEMENT

The authors declare that they have no known competing financial interests or personal relationships that could have appeared to influence the work reported in this paper.

## Supporting information


Figure S1.

## Data Availability

All input data and scripts for each analysis can be found in a permanent Dryad repository https://doi.org/10.5061/dryad.qz612jmqn.
