# Supplementary figures and images for: “Two zones and three centers” distribution and suitable areas shift of an evergreen oak in subtropical China under climate scenarios
Source: Ecol Evol. 2024 Sep 11;14(9):e70300. doi: 10.1002/ece3.70300 (PMC11390128; doi:10.1002/ece3.70300)

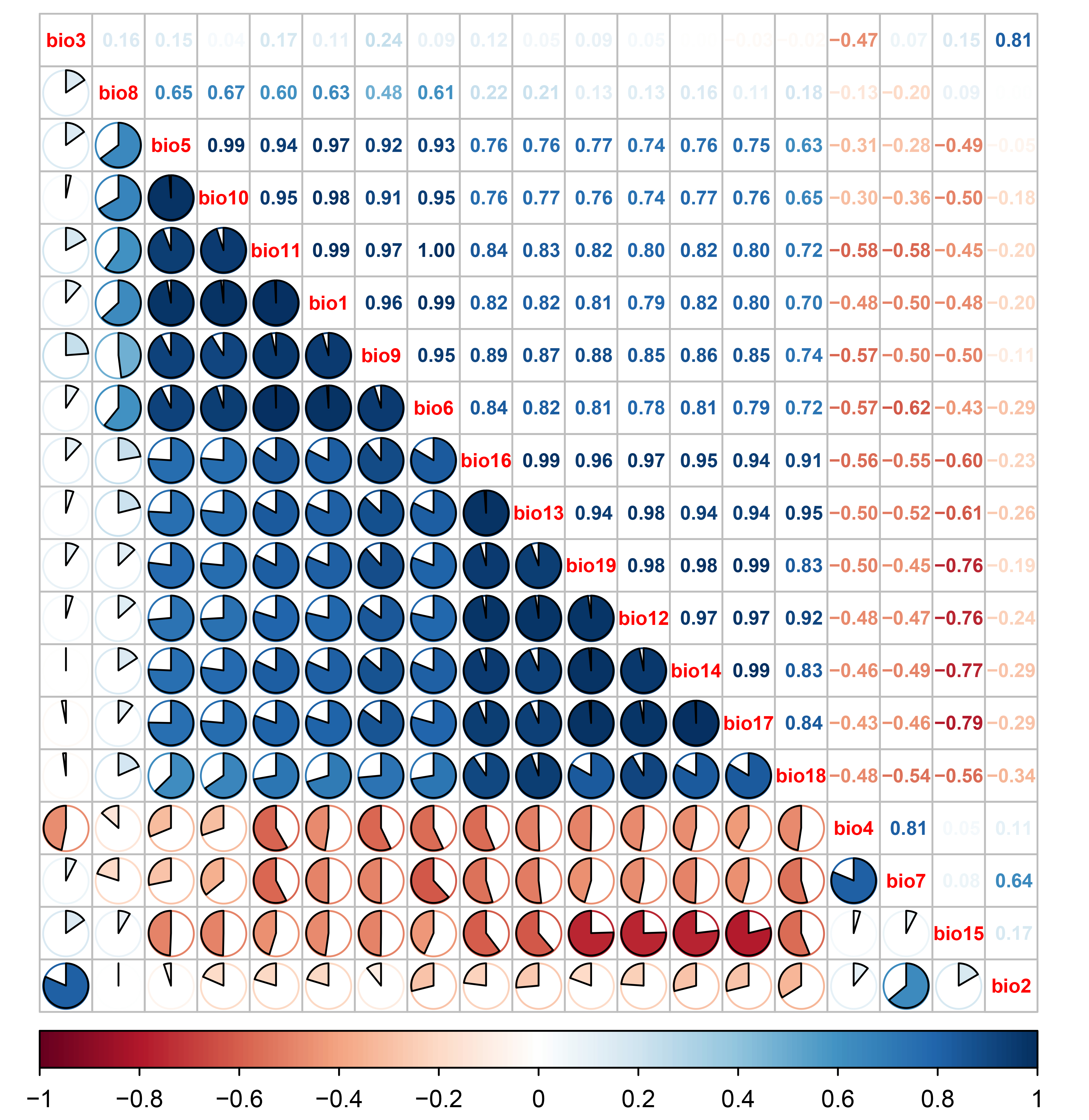

Supplement: Supplementary file 1 — Figure S1. [file ECE3-14-e70300-s001.jpg]
